# Supplementary material for: A novel transcriptional signature identifies T-cell infiltration in high-risk paediatric cancer
Source: Genome Med. 2023 Apr 3;15:20. doi: 10.1186/s13073-023-01170-x (PMC10071693; doi:10.1186/s13073-023-01170-x)
Supplement: Supplementary file 1 — Additional file 1: Table S1. Immune checkpoint and regulatory genes. Fig. S1. PRISM clinical trial study schema. Fig. S2. Cohort overview. Fig. S3. Deconvolution algorithms exhibit high concordance and an abundance of M2 macrophages in paediatric cancer. Fig. S4. Immunohistochemistry identifies paediatric patients with T-cell infiltrated tumours. Fig. S5. Prior treatment and steroid administration do not significantly affect T-cell infiltration. Fig. S6. Distribution of IPASS and T-cell clones are heterogenous across histologies. Fig. S7. IPASS correlations with additional markers of immune infiltration. [file 13073_2023_1170_MOESM1_ESM.pdf]

Table S1. Immune checkpoint and regulatory genes

ImmuneCheckpoint_RegulatoryGenes
CD8
CD200
CD27
CD274
CD276
CD40
CD70
CEACAM5
CEACAM6
CTLA4
EPHA2
ERBB2
FUT4
HAVCR2
HSPA5
ICOS
IDO1
KLRC1
LAG3
PDCD1LG2
TIGIT
TGFB1
TNFRSF18
TNFRSF4
TNFRSF9
VEGFA
VTCN1
IFNGR1
IFNGR2
IRF1
JAK1
JAK2
PTEN
STAT1
CSF1

Fig. S1. PRISM clinical trial study schema

Flow diagram of the PRISM clinical trial study schema (NCT03336931). Data utilised in this retrospective study was obtained from the enrolled patients who had whole genome and RNA sequencing (N=347) and for those patients with matching tumour tissue sections (N=78) to perform immunohistochemistry.

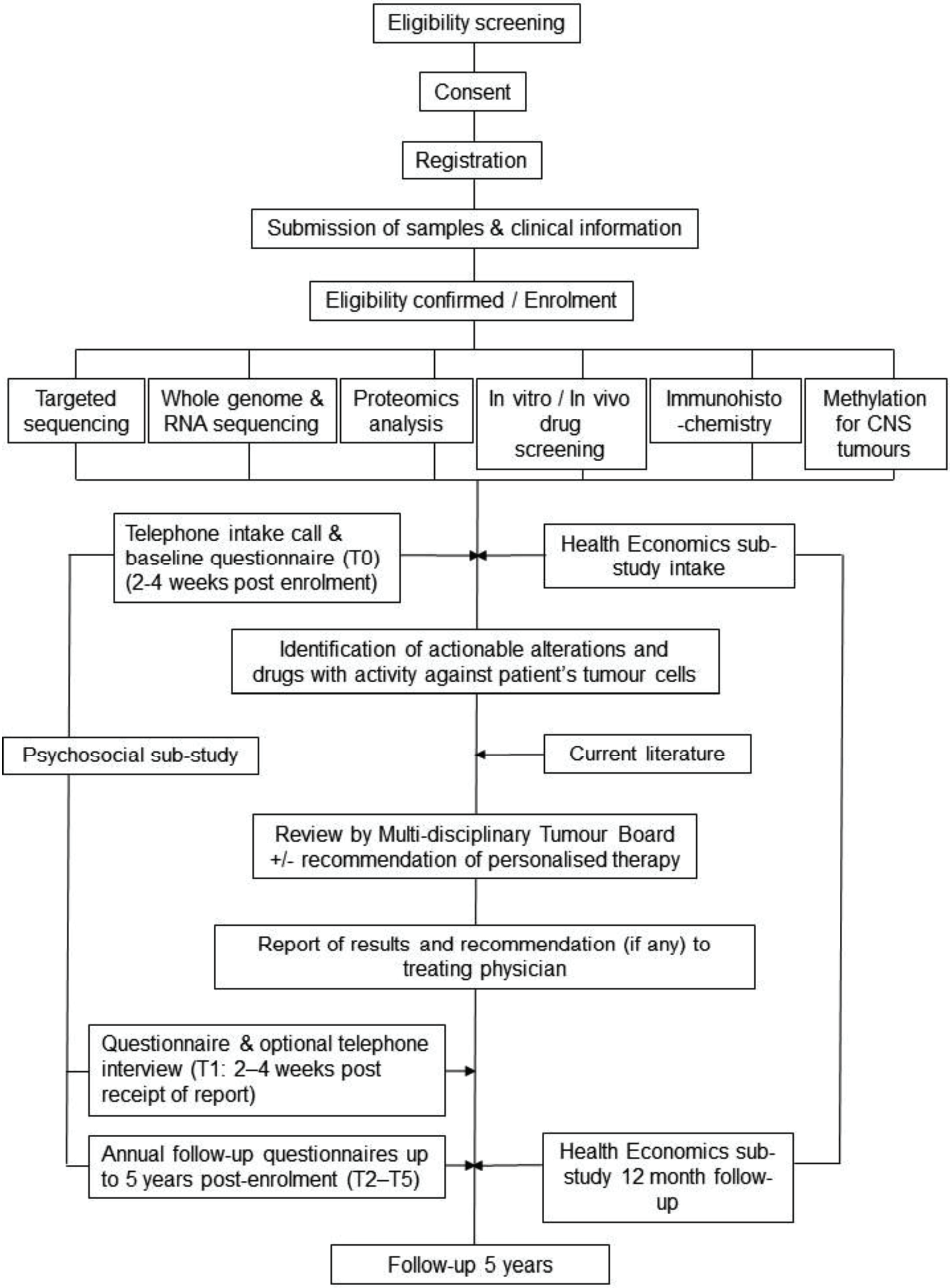
Eligibility screening
Consent
Registration
Submission of samples & clinical information
Eligibility confirmed / Enrolment
Targeted sequencing
Whole genome & RNA sequencing
Proteomics analysis
In vitro / In vivo drug screening
Immunohisto -chemistry
Methylation for CNS tumours
Telephone intake call & baseline questionnaire (T0) (2-4 weeks post enrolment)
Health Economics sub-study intake
Identification of actionable alterations and drugs with activity against patient's tumour cells
Psychosocial sub-study
Current literature
Review by Multi-disciplinary Tumour Board +/- recommendation of personalised therapy
Report of results and recommendation (if any) to treating physician
Questionnaire & optional telephone interview (T1: 2–4 weeks post receipt of report)
Annual follow-up questionnaires up to 5 years post-enrolment (T2–T5)
Health Economics sub-study 12 month follow-up
Follow-up 5 years

Fig. S2. Cohort Overview

Representation of the cohort consisting of 347 high-risk paediatric cancers who underwent RNA-seq. The cohort is highlighted by the frequency of samples, from the inner most ring to the outer ring by: CNS tumours (CNS), extracranial tumours and haematological malignancies (HM), further divided into histologies, stage of disease (diagnosis, refractory disease, relapse and secondary cancer), and immunohistochemistry (IHC) performed for samples highlighted in grey. Cancer histology key: acute myeloid leukemia (AML), B-precursor acute lymphoblastic leukemia (BALL), Juvenile myelomonocytic leukaemia (JMML), T-cell acute lymphoblastic leukemia (TALL), diffuse midline glioma (DMG), ependymoma (EPD), high grade glioma (HGG), medulloblastoma (MB), atypical teratoid rhabdoid tumour (ATRT), Ewing's sarcoma (EWS), malignant peripheral nerve sheath tumour (MPNST), neuroblastoma (NBL), osteosarcoma (OST), malignant rhabdoid tumour (MRT), rhabdomyosarcoma fusion negative (RMS FN), rhabdomyosarcoma fusion positive (RMS FP), and Wilms tumour (WT).

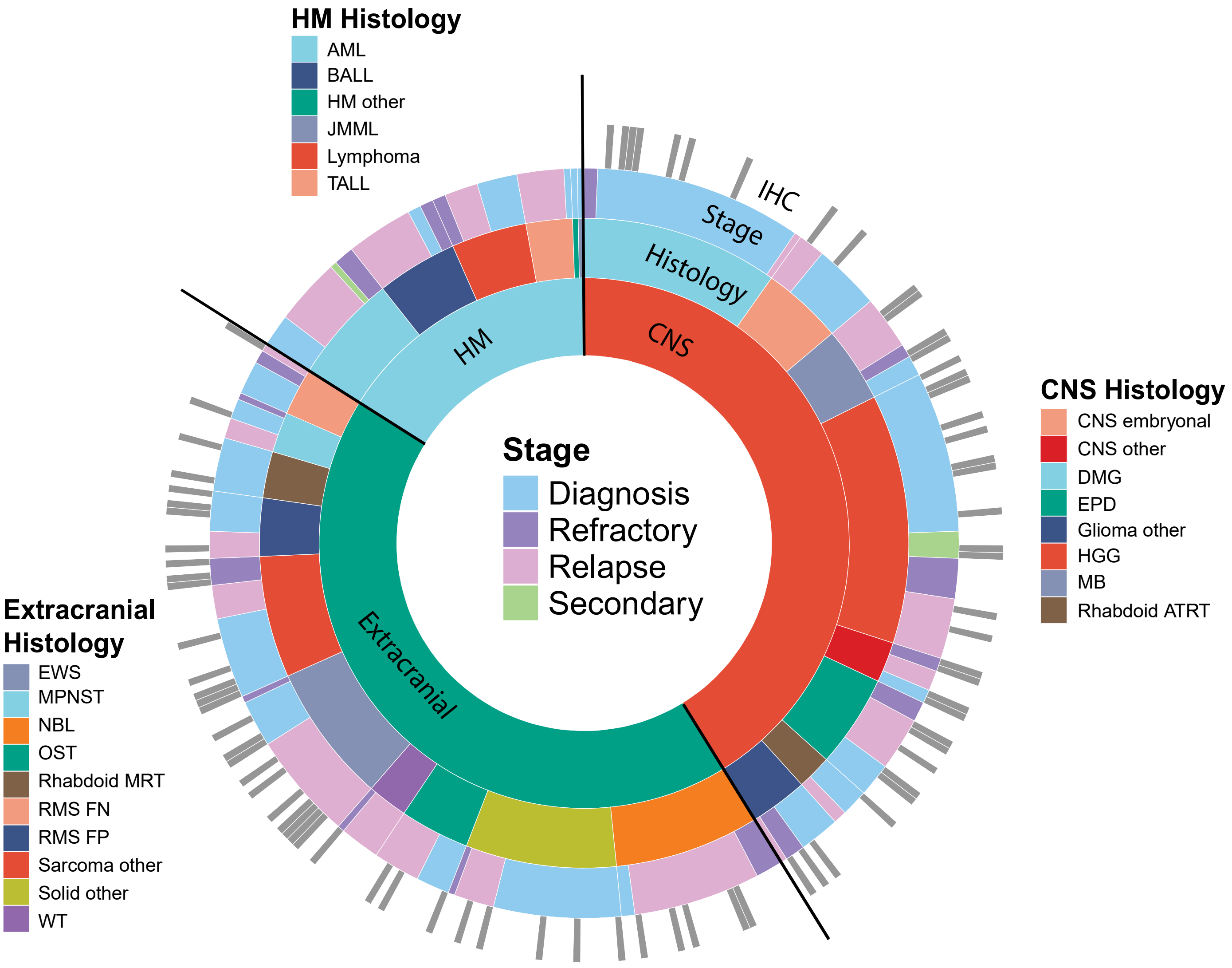

HM Histology
AML
BALL
HM other
JMML
Lymphoma
TALL
IHC
Stage
Histology
HM
CNS
Extracranial
Stage
Diagnosis
Refractory
Relapse
Secondary
CNS Histology
CNS embryonal
CNS other
DMG
EPD
Glioma other
HGG
MB
Rhabdoid ATRT
Extracranial Histology
EWS
MPNST
NBL
OST
Rhabdoid MRT
RMS FN
RMS FP
Sarcoma other
Solid other
WT

Fig. S3. Deconvolution algorithms exhibit high concordance and an abundance of M2 macrophages in paediatric cancer

Correlation of total CD8 T-cells between **(a)** CIBERSORTx (CSX) and quanTIseq, **(b)** CSX and MCP-counter (MCP) and **(c)** quanTIseq and MCP. Blue line is the correlation line of best fit. Proportion of all leukocytes (y-axis) for each patient (x-axis) separated by diagnosis in **(d)** CNS and **(e)** extracranial tumours.

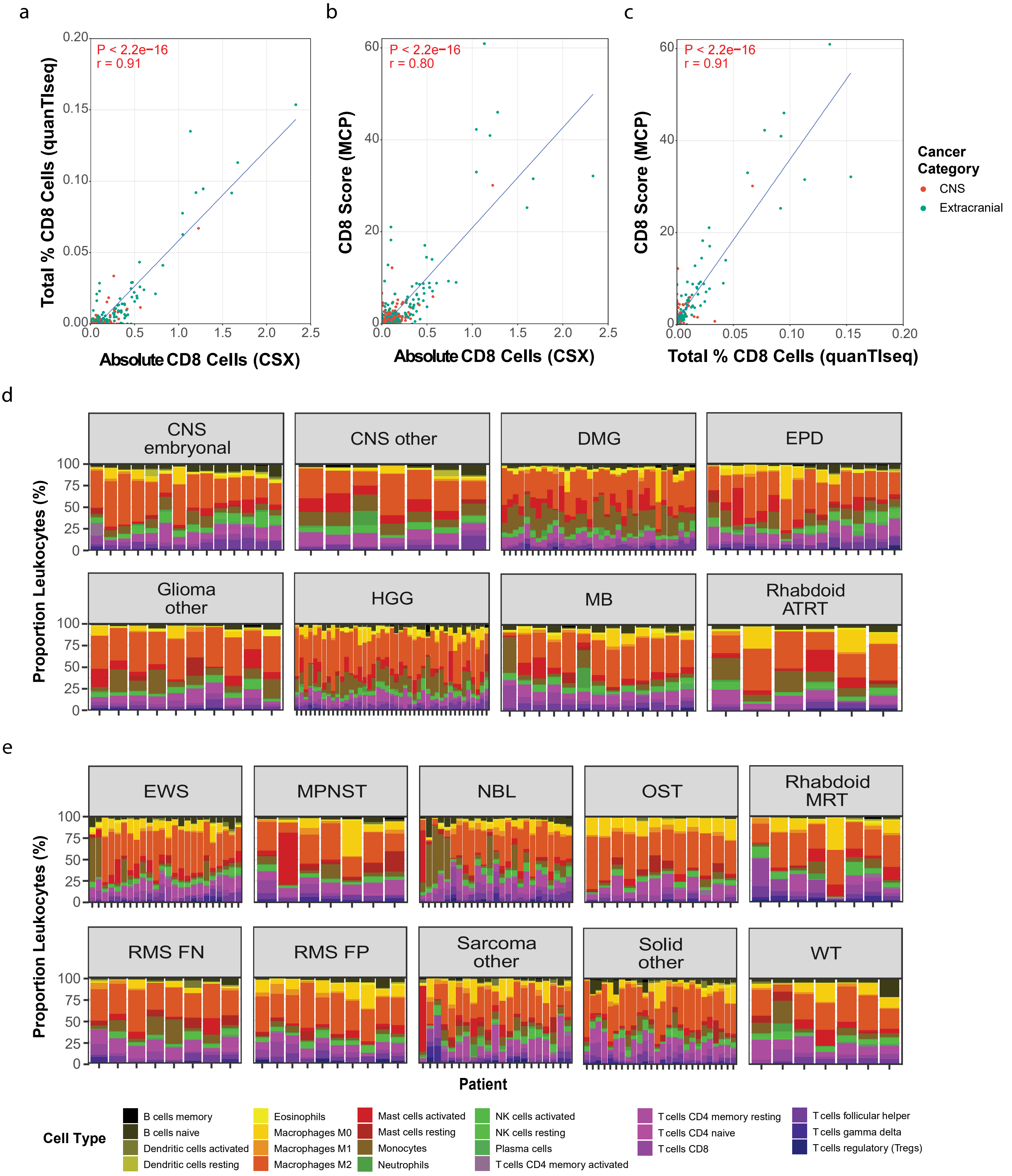
a
b
c
P < 2.2e−16
r = 0.91
Total % CD8 Cells (quanTIseq)
Absolute CD8 Cells (CSX)
P < 2.2e−16
r = 0.80
CD8 Score (MCP)
Absolute CD8 Cells (CSX)
P < 2.2e−16
r = 0.91
CD8 Score (MCP)
Total % CD8 Cells (quanTIseq)
Cancer Category
CNS
Extracranial
d
CNS embryonal
CNS other
DMG
EPD
Glioma other
HGG
MB
Rhabdoid ATRT
Proportion Leukocytes (%)
e
EWS
MPNST
NBL
OST
Rhabdoid MRT
RMS FN
RMS FP
Sarcoma other
Solid other
WT
Proportion Leukocytes (%)
Patient
Cell Type
B cells memory
B cells naive
Dendritic cells activated
Dendritic cells resting
Eosinophils
Macrophages M0
Macrophages M1
Macrophages M2
Mast cells activated
Mast cells resting
Monocytes
Neutrophils
NK cells activated
NK cells resting
Plasma cells
T cells CD4 memory activated
T cells CD4 memory resting
T cells CD4 naive
T cells CD8
T cells follicular helper
T cells gamma delta
T cells regulatory (Tregs)

Fig. S4. Immunohistochemistry identifies paediatric patients with T-cell infiltrated tumours

Representative IHC images of human tonsil, CNS tumours and extracranial tumours expressing high and low numbers of **(a)** $CD45^+$ cells, **(b)** $CD8^+$ T-cells and **(c)** $CD4^+$ T-cells. Correlation between number/mm^2 of $CD8^+$ T-cells by IHC compared to **(d)** total percent of CD8 T-cells by quanTIseq and **(e)** CD8 score by MCP-counter (MCP). **(f)** Absolute CD8 T-cells in CSX separated by IHC classification in extracranial tumours.

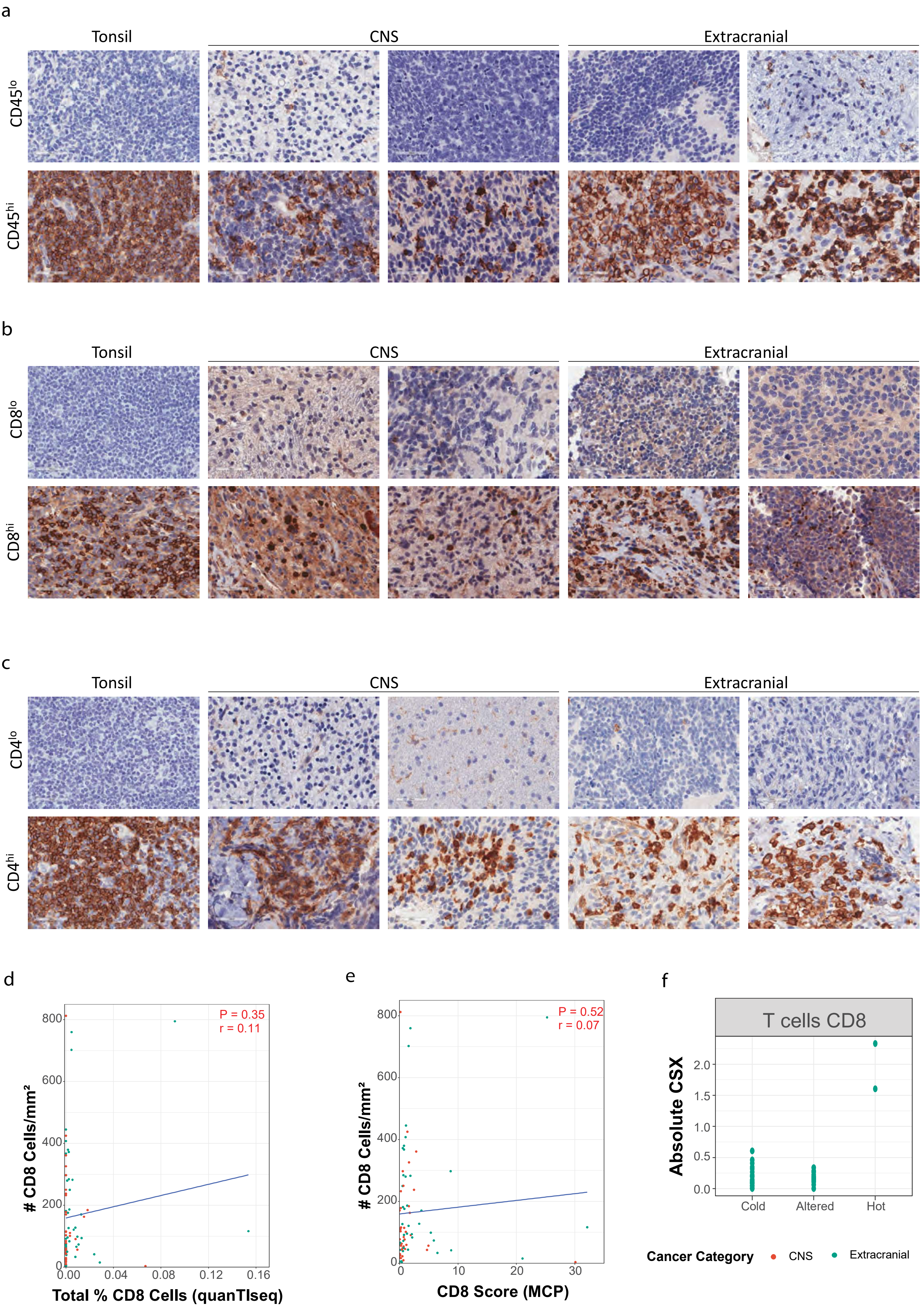

a
Tonsil
CNS
Extracranial
CD45lo
CD45hi
b
Tonsil
CNS
Extracranial
CD8lo
CD8hi
c
Tonsil
CNS
Extracranial
CD4lo
CD4hi
d
P = 0.35
r = 0.11
# CD8 Cells/mm²
Total % CD8 Cells (quanTIseq)
e
P = 0.52
r = 0.07
# CD8 Cells/mm²
CD8 Score (MCP)
f
T cells CD8
Absolute CSX
Cold
Altered
Hot
Cancer Category
CNS
Extracranial

Fig. S5. Prior treatment and steroid administration do not significantly affect T-cell infiltration

The number/mm^2 of **(a)** $CD45^+$, **(b)** $CD8^+$ and **(c)** $CD4^+$ cells in CNS (red) and extracranial (blue) tumours identified by IHC separated into yes or no having had received chemotherapy or radiation treatment within 42 days (n=76). The number/mm^2 of **(d)** $CD45^+$, **(e)** $CD8^+$ and **(f)** $CD4^+$ cells by IHC in CNS (red) tumours by IHC separated into yes or no having had received corticosteroid treatment within 7 days (n=37). Horizontal line is the median percent of cells and the ends of the box represent the upper and lower quartiles.

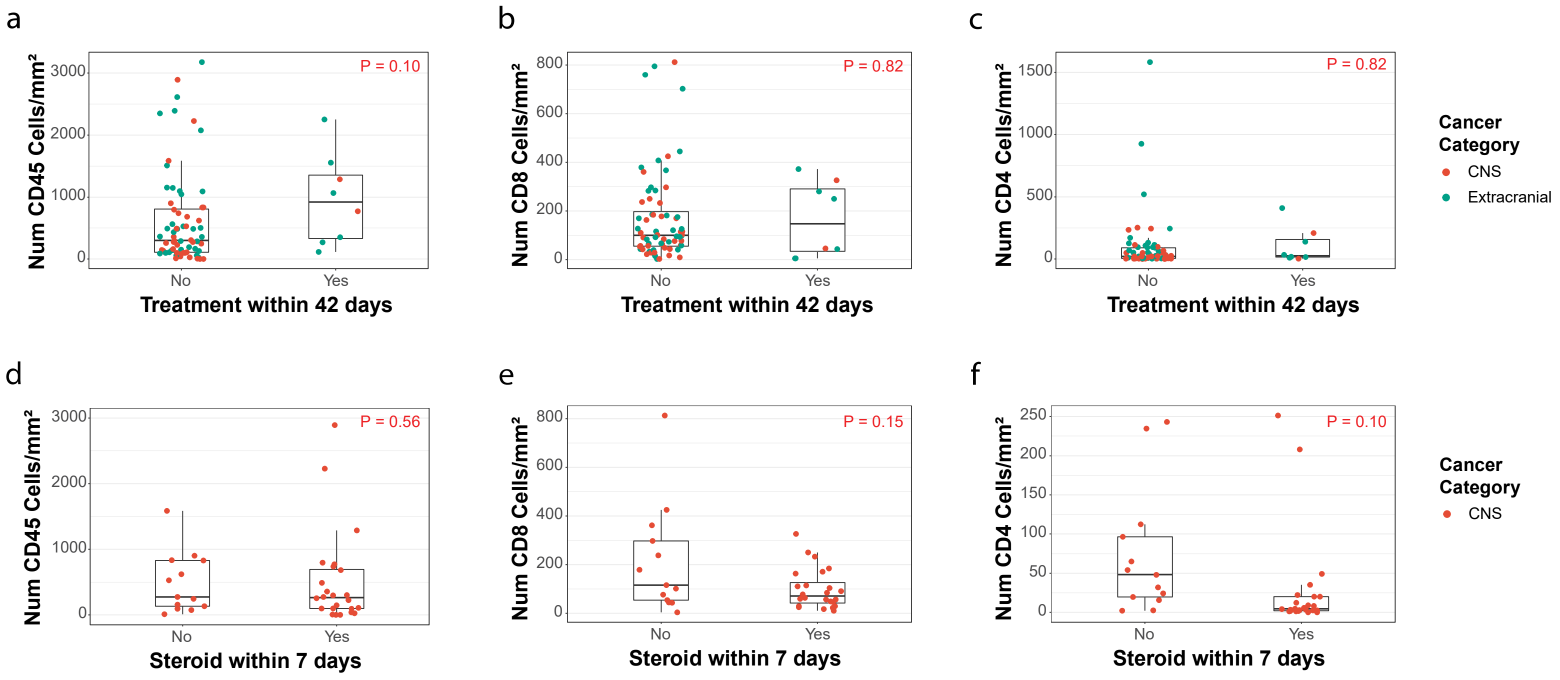

a
Num CD45 Cells/mm²
P = 0.10
No
Yes
Treatment within 42 days
b
Num CD8 Cells/mm²
P = 0.82
No
Yes
Treatment within 42 days
c
Num CD4 Cells/mm²
P = 0.82
No
Yes
Treatment within 42 days
Cancer Category
CNS
Extracranial
d
Num CD45 Cells/mm²
P = 0.56
No
Yes
Steroid within 7 days
e
Num CD8 Cells/mm²
P = 0.15
No
Yes
Steroid within 7 days
f
Num CD4 Cells/mm²
P = 0.10
No
Yes
Steroid within 7 days
Cancer Category
CNS

Fig. S6. Distribution of IPASS and T-cell clones are heterogeneous across histologies

Heatmap of the IPASS in **(a)** CNS tumours (N=143) and (**b**) extracranial tumours (N=148). Top annotation bar represents the cancer category, and second annotation bar represents treatment (within 42 days) (Dark grey; Yes, Grey; unknown, Light grey; No). The third annotation bar in a represents steroid administration (within 7 days) (Dark grey; Yes, Grey; unknown, Light grey; No, White; not applicable (as not a CNS tumour)). The fourth annotation bar in a and third in b is CD8 IHC classification (white are samples with no IHC data). The bottom annotation bar is the normalised IPASS score measured between 1 (green) and -1 (orange). **(c)** Heatmap of the IPASS extended to all samples in the validation cohort that had RNA-seq data from NCH (n=64) and ZERO (n=57). Top annotation bar represents the cancer category, second annotation is CD8 IHC classification (white are samples with no IHC data), and bottom annotation bar is the normalised IPASS score measured between 1 (green) and -1 (orange). Number of T-cell clones across **(d)** CNS and **(e)** extracranial tumours. Horizontal line is the median number of T-cell clones and the ends of the box represent the upper and lower quartiles. **(f)** Correlation between the IPASS and total number of T-cell clones (n=291). Blue line is correlation line of best fit.

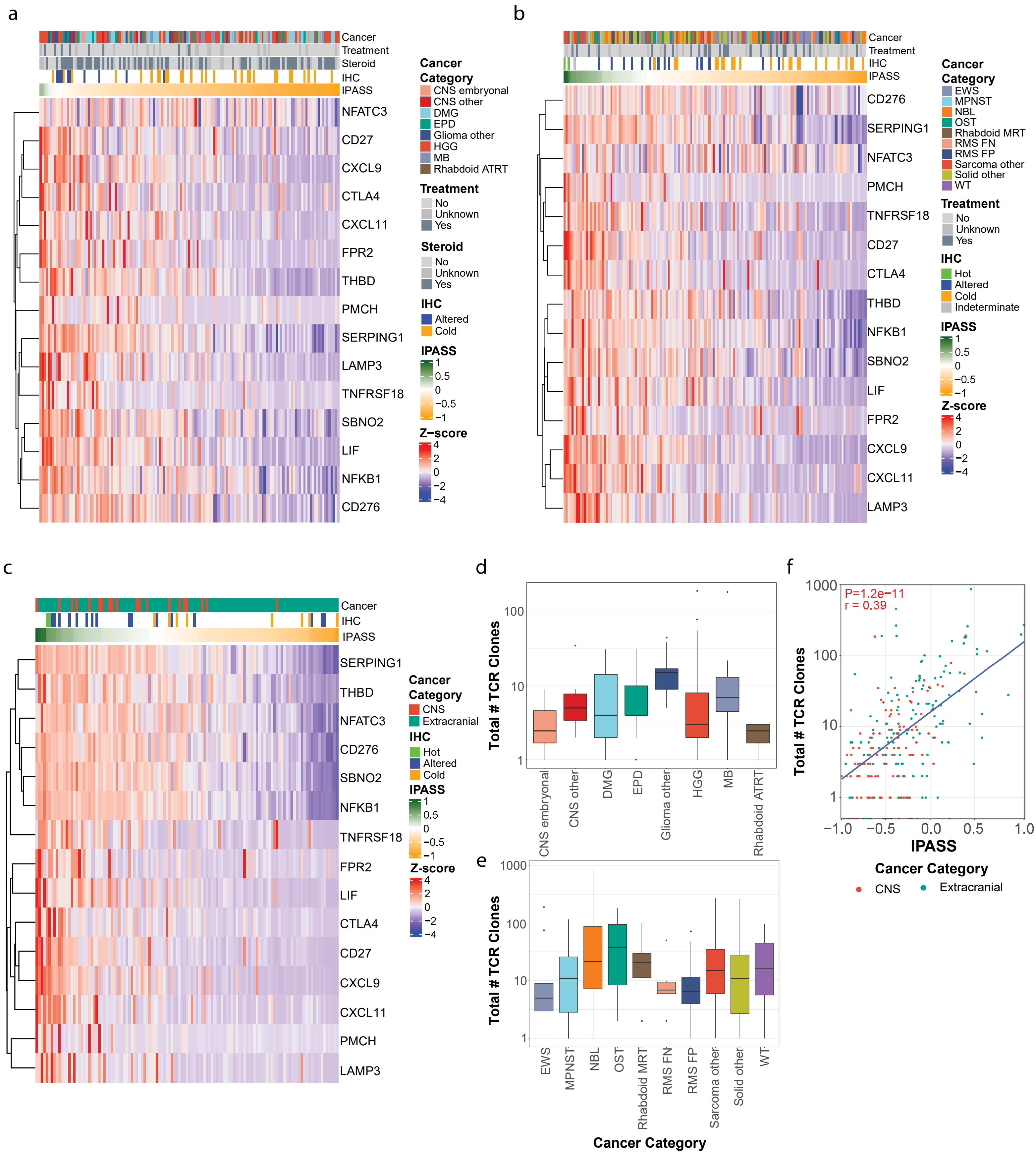

a
Cancer
Treatment
Steroid
IHC
IPASS
NFATC3
CD27
CXCL9
CTLA4
CXCL11
FPR2
THBD
PMCH
SERPING1
LAMP3
TNFRSF18
SBNO2
LIF
NFKB1
CD276
Cancer Category
CNS embryonal
CNS other
DMG
EPD
Glioma other
HGG
MB
Rhabdoid ATRT
Treatment
No
Unknown
Yes
Steroid
No
Unknown
Yes
IHC
Altered
Cold
IPASS
1
0.5
0
−0.5
−1
Z-score
4
2
0
−2
−4
b
Cancer
Treatment
IHC
IPASS
CD276
SERPING1
NFATC3
PMCH
TNFRSF18
CD27
CTLA4
THBD
NFKB1
SBNO2
LIF
FPR2
CXCL9
CXCL11
LAMP3
Cancer Category
EWS
MPNST
NBL
OST
Rhabdoid MRT
RMS FN
RMS FP
Sarcoma other
Solid other
WT
Treatment
No
Unknown
Yes
IHC
Hot
Altered
Cold
Indeterminate
IPASS
1
0.5
0
−0.5
−1
Z-score
4
2
0
−2
−4
c
Cancer
IHC
IPASS
SERPING1
THBD
NFATC3
CD276
SBNO2
NFKB1
TNFRSF18
FPR2
LIF
CTLA4
CD27
CXCL9
CXCL11
PMCH
LAMP3
Cancer Category
CNS
Extracranial
IHC
Hot
Altered
Cold
IPASS
1
0.5
0
−0.5
−1
Z-score
4
2
0
−2
−4
d
Total # TCR Clones
100
10
1
CNS embryonal
CNS other
DMG
EPD
Glioma other
HGG
MB
Rhabdoid ATRT
e
Total # TCR Clones
1000
100
10
1
EWS
MPNST
NBL
OST
Rhabdoid MRT
RMS FN
RMS FP
Sarcoma other
Solid other
WT
Cancer Category
f
P=1.2e−11
r = 0.39
Total # TCR Clones
1000
100
10
1
−1.0
−0.5
0.0
0.5
1.0
IPASS
Cancer Category
CNS
Extracranial

Fig. S7. IPASS correlations with additional markers of immune infiltration

Number of neoantigens distributed across **(a)** CNS and **(b)** extracranial tumours. The red line represents the median number of neoantigens. Correlation between **(c)** total number of neoantigens and mutations/Mb (coding missense SNPs), **(d)** IPASS and mutations/Mb (coding missense SNPs), **(e)** IPASS and total number of neoantigens, and **(f)** IPASS and tumour purity (n=291). Blue line is correlation line of best fit. Heatmap of all immune checkpoint genes examined in **(g)** CNS tumours (n=143) and **(h)** extracranial tumours (n=148). Top annotation bar represents the cancer category, second annotation is CD8 IHC classification (white are samples with no IHC data) and third annotation bar is the IPASS score measured between 1 (green) and -1 (orange). Bottom annotation bar from top to bottom represents the number of T-cell receptor (TCR) clones, tumour purity (percentage of malignant cells), tumour mutation burden (TMB) and neoantigen load. Proportion of tumours broken into diagnosis assigned to their given dominant immune archetype in **(i)** CNS and **(j)** extracranial tumours. Archetype key: dendritic cells (DC), immune stromal rich (ISR), immune rich (IR), classical DC (cDC), and immune desert (ID).

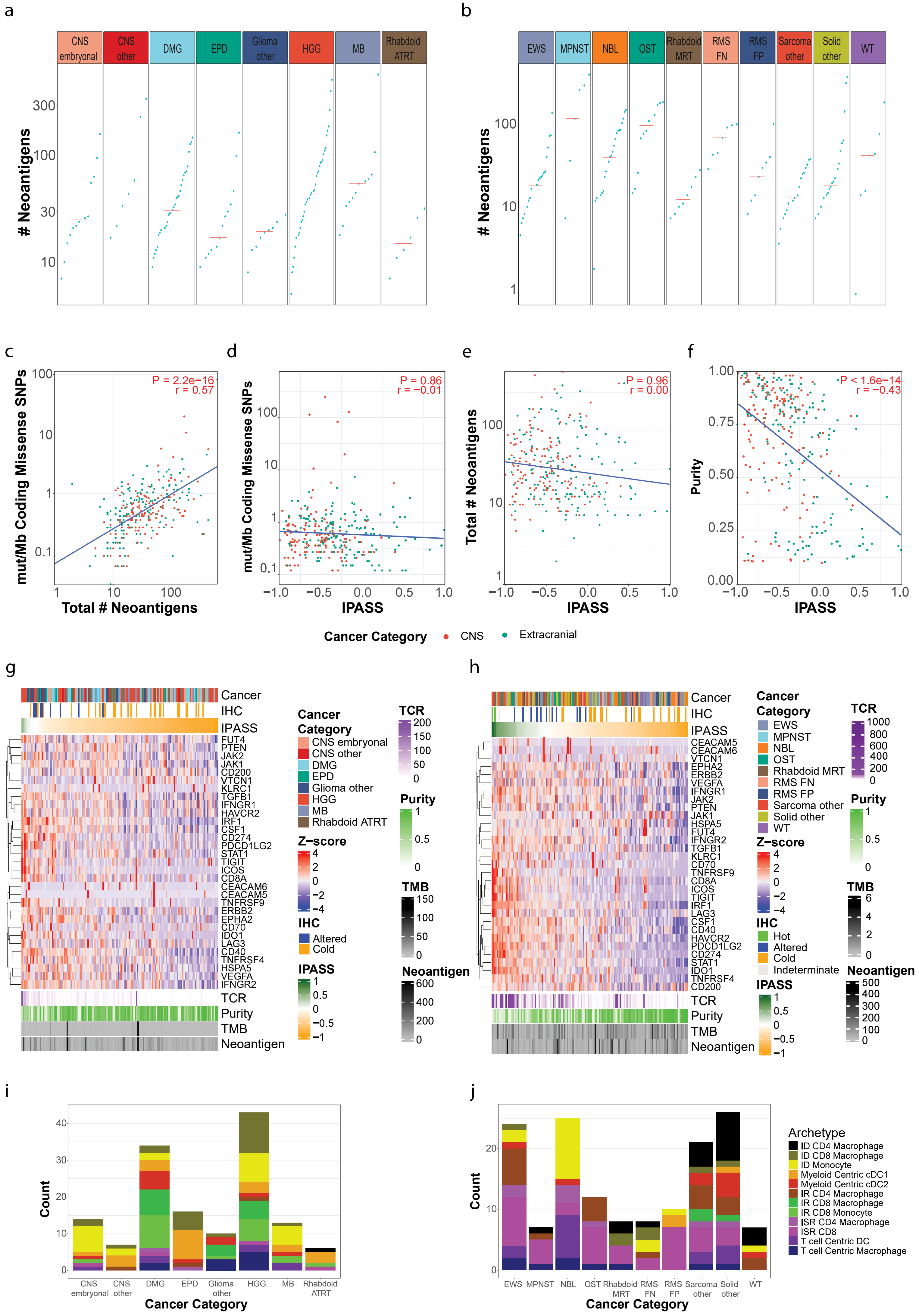
a
CNS embryonal
CNS other
DMG
EPD
Glioma other
HGG
MB
Rhabdoid ATRT
Neoantigens
b
EWS
MPNST
NBL
OST
Rhabdoid MRT
RMS FN
RMS FP
Sarcoma other
Solid other
WT
Neoantigens
c
P = 2.2e−16
r = 0.57
mut/Mb Coding Missense SNPs
Total # Neoantigens
d
P = 0.86
r = −0.01
mut/Mb Coding Missense SNPs
IPASS
e
P = 0.96
r = 0.00
Total # Neoantigens
IPASS
f
P < 1.6e−14
r = −0.43
Purity
IPASS
Cancer Category
CNS
Extracranial
g
Cancer
IHC
IPASS
FUT4
PTEN
JAK2
JAK1
CD200
VTCN1
KLRC1
TGFB1
IFNGR1
HAVCR2
IRF1
CSF1
CD274
PDCD1LG2
STAT1
TIGIT
ICOS
CD8A
CEACAM6
CEACAM5
TNFRSF9
ERBB2
EPHA2
CD70
IDO1
LAG3
CD40
TNFRSF4
HSPA5
VEGFA
IFNGR2
TCR
Purity
TMB
Neoantigen
Cancer Category
CNS embryonal
CNS other
DMG
EPD
Glioma other
HGG
MB
Rhabdoid ATRT
Z−score
IHC
Altered
Cold
IPASS
TCR
Purity
TMB
Neoantigen
h
Cancer
IHC
IPASS
CEACAM5
CEACAM6
VTCN1
EPHA2
ERBB2
VEGFA
IFNGR1
JAK2
PTEN
JAK1
HSPA5
FUT4
IFNGR2
TGFB1
KLRC1
CD70
TNFRSF9
CD8A
ICOS
TIGIT
IRF1
LAG3
CSF1
CD40
HAVCR2
PDCD1LG2
CD274
STAT1
IDO1
TNFRSF4
CD200
TCR
Purity
TMB
Neoantigen
Cancer Category
EWS
MPNST
NBL
OST
Rhabdoid MRT
RMS FN
RMS FP
Sarcoma other
Solid other
WT
Z−score
IHC
Hot
Altered
Cold
Indeterminate
IPASS
TCR
Purity
TMB
Neoantigen
i
Count
Cancer Category
j
Count
Cancer Category
Archetype
ID CD4 Macrophage
ID CD8 Macrophage
ID Monocyte
Myeloid Centric cDC1
Myeloid Centric cDC2
IR CD4 Macrophage
IR CD8 Macrophage
IR CD8 Monocyte
ISR CD4 Macrophage
ISR CD8
T cell Centric DC
T cell Centric Macrophage
